# Medical Manners and Mystery

**Published:** 1921-09-17

**Authors:** 


### Letters to a Nurse.

### I.—MEDICAL MANNERS AND MYSTERY.

Dear Barbara,—The other day I read a letter somewhere—I forget where—from a disgruntled patient. He complained that his doctor put on airs, played the high-brow game, declined to explain anything at all, etc., etc.

Well, my sympathies are with the patient. After all, he *is* the patient. His trouble is his own; it is not a private secret belonging to the doctor. And he has a perfect right to know all that the doctor has to tell. Mind you, I am taking it for granted that the patient is a sane person. If he is a half-baked ass the doctor, *perhaps*, is justified in treating him as a half-baked ass. But . . . !

See here—you are going out into the world as a private nurse. You will meet many patients and many doctors. You will have to learn for yourself how you stand with both; but you can take this from me for what it is worth —a doctor who is unduly mysterious, a doctor who will persist in keeping all he knows to himself, knows precious little. The doctor to trust is the man who is perfectly frank, even if his manner is a trifle brusque. The gentleman who washes his hands with invisible soap (all right—I know that's a *cliché*, so you needn't bother to say so) and departs in a blaze of perfect manners and mystery, is a doctor to distrust. The chances are that he says nothing because he has nothing to say—because he is ignorant.

I know a doctor who has the most charming manners imaginable. His patients love him. "He is so sympathetic." Perhaps he is; but he never lets his sympathy carry him to the length of sharing the premature grave he has prepared for his patients. They suffer and die before their time as a result of his ministrations, and they worship him! He is the one doctor in the world for them. But I wonder what would happen if he were to bump up against my disgruntled friend. Perhaps nothing, perhaps . . . ! But I don't think he is the sort of man my disgruntled friend was writing about. Probably he had in his mind that particularly unpleasant brand of young doctor who is stuffed full with the unwisdom of the schools, but has no worldly experience; who is contemptuous of everyone older than himself—particularly his patient and his patient's friends.

My dear—young nurses sometimes are equally insufferable. They regard each patient merely as a case, and the patient's friends as a collection of interfering fools. Don't fall into that error. A patient, and the patient's friends, have a right to all the information and all the sympathy you and the doctor (however young) may have at your disposal. Be frank. Respect the doctor who is frank. Never try the mystery game. We have got past the mediæval and Victorian ages.

Your affectionate

Uncle Luke.

